# Enhancing Patient Throughput Through Front-end Redesign and Alternate Care Space Implementation in a Pediatric Emergency Department: A Quality Improvement Initiative

**DOI:** 10.1097/pq9.0000000000000923

**Published:** 2026-09-28

**Authors:** Cortney C. Braund, Bernadette K. Johnson, Shaela Moen, Sandra Spencer, Travis Justilian, Suzanne Sternberg, Kristin Vickrey, Matthew Cerchie, Candise Dunham, Nidhya Navanandan

**Affiliations:** From the Department of Pediatrics, Section of Emergency Medicine, Children’s Hospital Colorado, University of Colorado, Aurora, Colo.

## Abstract

**Introduction:**

Emergency department (ED) crowding remains a persistent national challenge, contributing to delays in care, increased length of stay (LOS), and higher rates of patients leaving without being seen (LWBS). Front-end strategies improve throughput and reduce LWBS by enabling earlier assessment and disposition. Building on prior efforts, this quality improvement initiative aimed to enhance patient flow for low-acuity pediatric patients by leveraging alternative care spaces to reduce roomed-to-disposition time, LOS, and LWBS while preserving capacity for higher acuity care.

**Methods:**

This initiative occurred in a high-volume pediatric ED using a front-end, physician-driven “Intake” model. To improve efficiency and throughput, the team introduced an alternate care space. Additional interventions included role clarification, workflow standardization, and targeted education for physicians and staff to reduce roomed-to-disposition time for Intake patients by 10% within 4 months.

**Results:**

Roomed-to-disposition time for all Intake patients decreased by 15% (from 88 to 75 min), whereas a specific group of low-intervention patients experienced a 49% reduction. LOS for all Intake patients decreased by 30%, and by 48% for low-intervention patients (from 286 to 148 min). Additional improvements included a 49% reduction in LWBS and a 37% decrease in arrival-to-provider time.

**Conclusions:**

Our quality improvement initiative demonstrated that a thoughtful reorganization of existing ED Intake processes, without additional staffing or physical expansion, can meaningfully improve patient throughput, provider efficiency, and overall ED performance.

## INTRODUCTION

For more than 3 decades, crowding has been one of the most persistent challenges for emergency departments (EDs) across the nation. Crowding occurs when the demand for emergency services outstrips available resources to provide care. This creates constant tension between treating critically ill patients and managing high patient volumes. Delays in patient evaluation can jeopardize safety, as prolonged wait times may lead to worsening conditions, delayed treatments, and missed diagnoses. Moreover, longer wait times increase the likelihood of patients leaving without being seen (LWBS), further compounding the problem.^1,2^ During the respiratory surge of 2022, our local LWBS rates more than tripled and remained consistently elevated above baseline in the years that followed. This significant challenge compelled us to urgently seek and implement solutions to increase throughput and better manage patient flow, thereby improving care delivery.

Although EDs have limited control over the number or acuity of arriving patients, or the pace at which patients are transferred or admitted, they can improve patient throughput efficiency within the department through various workflow enhancements.^3^ Previous studies have demonstrated that implementing nursing protocols and initiating diagnostic testing during triage can reduce ED length of stay (LOS) and improve patient throughput.^4,5^ In 2023, the American Academy of Pediatrics updated its report and best practices on crowding in the ED, highlighting throughput solutions, including early front-end provider evaluation and split-flow processes that streamline patient flow for specific chief complaints and acuities after triage.^6^

Multiple studies demonstrate that improved front-end strategies minimize wait times, decrease ED LOS, and improve patient satisfaction. A 2020 systematic review concluded that, in addition to optimizing split-flow processes, shifting physician–patient contact to the earliest possible stage is a highly effective strategy for enhancing flow.^7^ One strategy involves adding a physician or advanced practice provider alongside a registered nurse (RN), as a “provider-in-triage” (PIT). This provider’s role is often to conduct a brief initial assessment or medical screening examination and to begin any necessary testing or simple treatment directly in the triage area when the main ED space is unavailable.^8^ Introducing a PIT results in decreased LWBS; however, debate remains over the best way to leverage the provider to affect throughput significantly.^9,10^ Other institutions have advanced the PIT concept by implementing a physician-driven front-end process. This model removes traditional nurse-based triage and places an attending physician at the forefront of the ED, supported by a dedicated team, to more thoroughly assess, treat, and determine patient disposition early in their visit.^11,12^

This institution developed a front-end, attending-driven process called “physician in Intake” in 2016. This change resulted in improved LWBS rates and door-to-provider times.^13^ In 2024, Lam et al^14^ attempted to further improve the LOS and discharge efficiency for low-acuity patients seen in Intake who required only brief intervention. This work redefined the criteria for these low-intervention patients and provided staff and provider education to achieve a 1-hour discharge. Although there was improvement, it was modest and underscored the need for further process enhancements to increase patient throughput.^14^ Here, we describe a quality improvement initiative aimed at optimizing patient flow for patients evaluated in Intake by leveraging alternative care spaces within the ED front end to decrease roomed-to-disposition time while preserving capacity for high-acuity patients in the main ED care area. Our primary aim was to reduce roomed-to-disposition time for Intake patients by 10% within 4 months.

## METHODS

### Context

We conducted this quality improvement initiative in a quaternary care, level I trauma center pediatric ED that sees approximately 70,000 annual visits. This institution operates a physician-driven front-end process, “Intake,” using 4 rapid assessment rooms to evaluate patients with Emergency Severity Index acuity levels 3–5 who meet this institution’s predefined Intake criteria.^13^ Intake operates year-round, from 11 am to 11 pm, and evaluates approximately 15,000 patients annually.

Before this initiative, following Intake evaluation, patients were either discharged or recommended for ongoing care in the main area of the ED. Patients anticipated to require 1 hour or less of additional care were designated as “Supertrack,” whereas those requiring more extensive evaluation were assigned as “main ED” patients. Before this intervention, both groups completed their care in the main ED treatment rooms, with a handoff to a new provider regardless of designation.

### Interventions

#### Intake and Front-end Kaizen in March of 2024

In March 2024, a multidisciplinary team conducted an intensive process investigation and project planning meeting, modeled after a Kaizen event. The team evaluated Intake workflows and identified opportunities for improvement. Participants included RNs, Pediatric Emergency Medicine physicians, emergency medical technicians (EMTs), scribes, and registration staff. The team initially intended to use the DMAIC (Define, Measure, Analyze, Improve, and Control) methodology for this problem, as they hypothesized that they could adjust and improve existing elements of the front-end Intake processes. DMAIC is a structured, data-driven methodology and a core problem-solving framework. However, during the process investigation, our mapping of the current state identified multiple areas for improvement and considerable variation across all processes. The team, therefore, determined that a Design for Six Sigma methodology would be more appropriate as we redesigned and created new processes within the front-end and Intake area of the ED.

A key driver diagram was developed after the process mapping and guided the identification of targeted interventions (Fig. 1). The team identified 5 essential elements to guide the redesign: (1) efficient patient throughput; (2) defined care spaces; (3) appropriate patient selection and resource allocation; (4) clear roles and accountability; and (5) consistent performance during peak demand. Following the Kaizen, a focused stakeholder group identified targeted interventions to address these elements in the redesign of Intake.

**Fig. 1.**
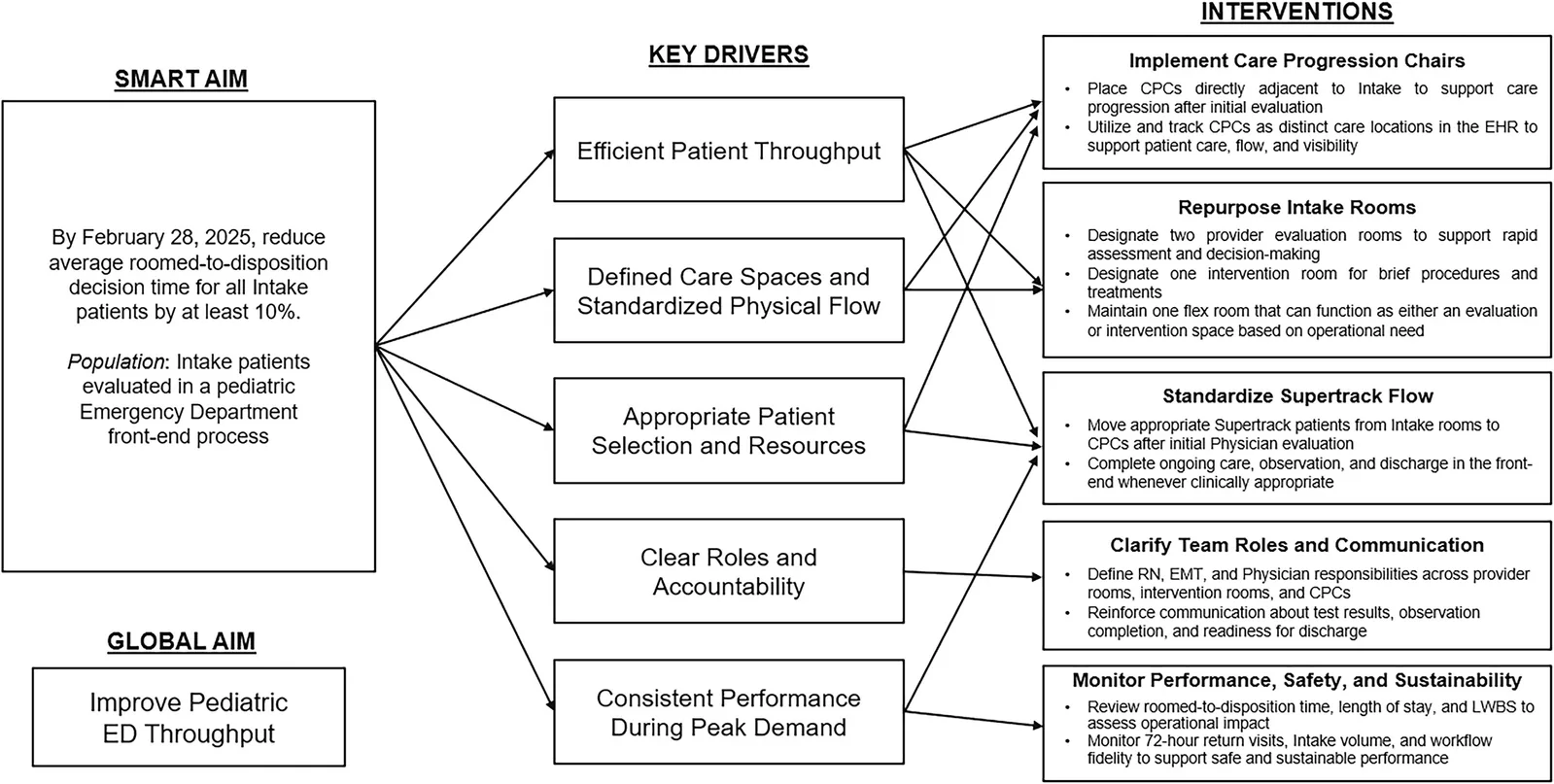
Key driver diagram.

#### Implementation of New Patient Flow Processes

Data review identified inefficiencies in Supertrack patient throughput, with patients spending an average of 64 minutes in their first Intake room and experiencing a total LOS of 213 minutes. Given this clearly defined yet inefficient cohort, interventions focused largely on Supertrack patients (Table 1).

**Table 1.** Supertrack Criteria

Intervention	Indication
Albuterol-metered dose inhaler	Mild asthma exacerbation not requiring additional asthma management adjuncts
Antihistamine	Pruritic rashes without other signs of anaphylaxis
Ear irrigation	Impacted cerumen precluding an ear examination to diagnose acute otitis media
Fluorescein eye examination	Suspected corneal abrasions in cooperative patients
Nasal suctioning	Nasal congestion in well-appearing patients without hypoxia or increased work of breathing
Specialty phone consultation	Subspecialty concerns that require only a single phone (not in-person) consultation
Urinalysis (obtained via catheter or clean catch)	Suspected urinary tract infection
Zofran and/or oral rehydration therapy	Mild dehydration and oral intolerance from viral gastroenteritis
X-ray imaging	Minor traumatic injuries without gross deformity

Patients evaluated in Intake who require 1 or 2 of the listed interventions for the specified indications qualify as Supertrack patients.

The team developed an ideal-state process map to streamline patient flow through Intake and to support the primary intervention of completing patient care in the newly implemented care progression chairs (CPCs), placed directly adjacent to the Intake area (Fig. 2). This intervention relocated Supertrack patient care from the main ED area to the CPCs. Intake rooms were repurposed to support specific functions: 2 rooms for provider evaluation, 1 intervention room for brief treatments, and 1 “flex” room. The flex room could serve as either an intervention room or a provider evaluation room, depending on operational needs. Following initial evaluation, we transitioned Supertrack patients directly from Intake rooms to CPCs whenever possible, reserving intervention rooms for patients requiring brief procedures.

**Fig. 2.**
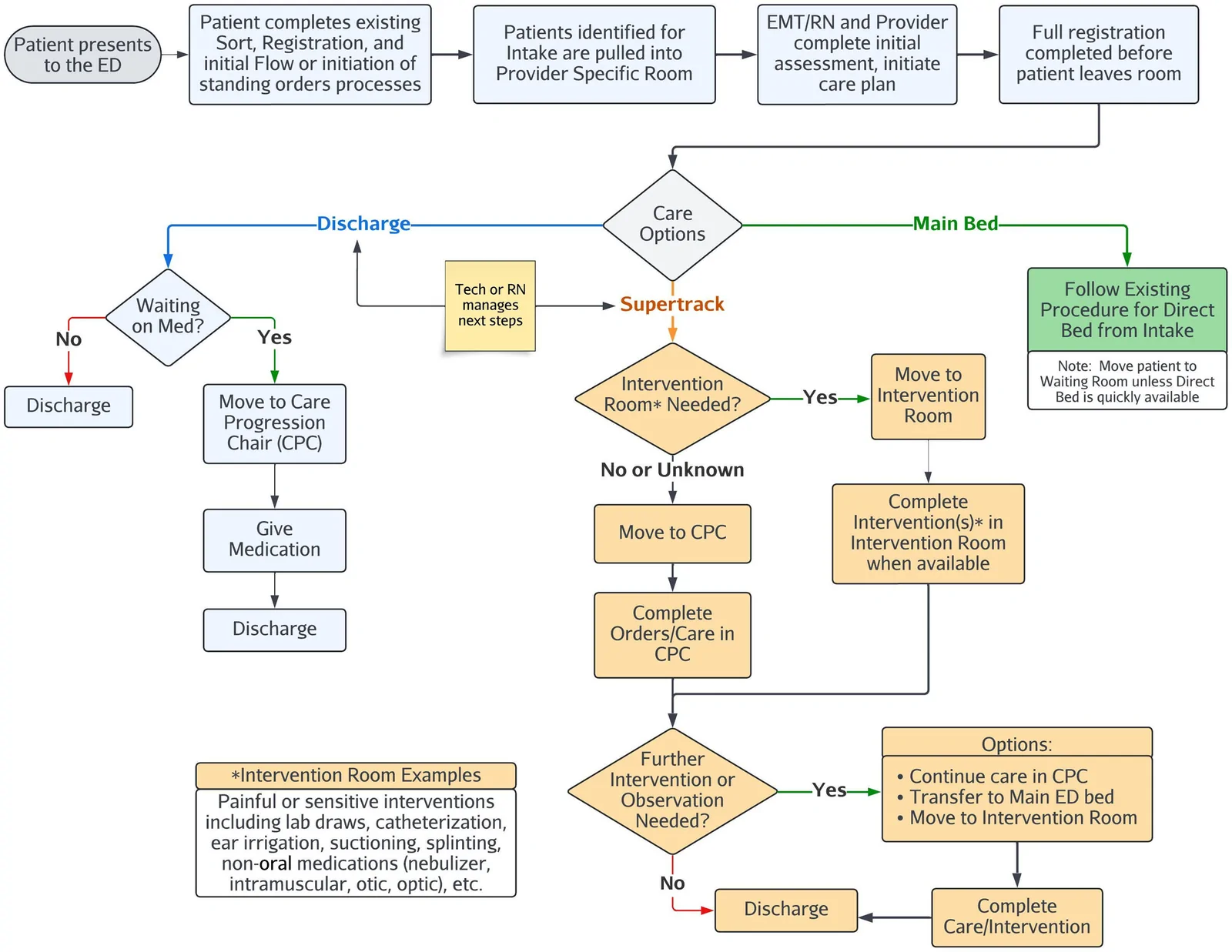
Ideal-state process map.

Each CPC unit consists of 3 seats, accommodating 1 patient and 2 family members. They are physically designated and tracked in the electronic health record (Epic Systems, Verona, WI). Care in CPCs remained under the Intake physician, who completed diagnostic evaluations, medication administration, observation, and discharge at the front end without occupying traditional ED rooms.

#### Role Definition

To support the revised workflow, we refined the EMT, RN, and physician roles within Intake and standardized them to align with care completion in the CPCs. Clear role descriptions outlined responsibilities for patient movement, monitoring, and communication across Intake provider rooms, intervention rooms, and CPCs. We communicated role expectations and workflow changes through multidisciplinary meetings and brief, role-specific educational materials. Additionally, we conducted targeted, just-in-time education throughout implementation.

### Project Aim and Core Measures

The project aimed to standardize Intake workflows to reduce process variation. We implemented CPCs as an alternative front-end care space to enable the completion of care for appropriate patients. We aimed to achieve a 10% reduction in average roomed-to-disposition decision time for all Intake patients within 4 months (from 88 to 79 min).

The primary outcome measure was roomed-to-disposition time for all Intake patients. We defined this as the interval from initial room assignment to the placement of a discharge or admission order in the electronic health record. This measure reflects front-end workflow efficiency more directly than total ED LOS. Secondary outcome measures included roomed-to-disposition time and LOS specifically for Supertrack patients. Additional measures included arrival-to-provider time, LOS, and the percentage of LWBS across all ED patients.

The primary process measure was time in the first room. We defined this as the interval from the initial room assignment to transfer to an alternate care space (including CPCs) or to the entry of a disposition order. We chose this measure because it reflects room turnover and the early transition of appropriate patients to CPCs, with a target of a 20% reduction from 61 to 49 minutes. Balancing measures included (1) 72-hour return ED visits for Supertrack patients and (2) the proportion of ED patients evaluated through Intake.

### Analysis

Given the seasonal variability in ED arrivals, the team selected the previous respiratory season as our baseline comparison period. This approach ensured that we compared time periods with similar patient volumes, focusing specifically on respiratory seasons rather than the entire calendar year. Before testing for statistical significance, data were categorized into the overall population, the Intake population, and the Intake Supertrack population, and assessed for normality using the Anderson-Darling normality tests. For normally distributed data, an iterative series of 1-sample *t* tests determined the measurement level at which statistically significant improvement could be demonstrated. For nonnormally distributed data, the team used the sign test for medians. Once the team established significance thresholds, process capability analysis was performed to define the most realistic, statistically significant performance goals. The team repeated this entire process 2 months into the intervention to verify that statistically significant improvements and process capability were sustained and feasible.

Data were then extracted from Epic’s SlicerDicer data model, which aggregates data points within Epic for further analysis. Filters were applied to isolate outcome and process data, specifically roomed-to-disposition time (outcome) and time in the first room (process), for the target patient populations. Following data extraction, the team used Minitab Statistical Software to generate statistical process control charts, specifically X-bar S charts, because the data were continuous and organized into daily subgroups with multiple encounter-level observations per day. We used X-bar charts to evaluate changes in the daily subgroup means for outcome and process measures, and S charts to assess within-day variation across observations within each subgroup. We evaluated the charts for evidence of process change using Nelson’s rules, with 8 consecutive points above or below the centerline indicating a potential process shift.^15^

### Ethical Considerations

This study was deemed not to constitute human subjects research by the institutional quality improvement review panel. There were no conflicts of interest or ethical concerns.

## RESULTS

During the intervention period (October 22, 2024, to February 28, 2025), we evaluated 27,809 patients at this pediatric ED, specifically 5,618 (18%) in Intake. Of the patients seen in Intake, we designated 729 as Supertrack. During the comparison period (October 22, 2023, to February 28, 2024), we evaluated 27,964 patients, with 5,517 (19%) seen in Intake and 293 designated as Supertrack. Although the proportion of patients evaluated in Intake remained stable, the proportion designated as Supertrack increased from 5% to 13% during the intervention period. Supertrack patients primarily completed care in the CPCs, consistent with the intended workflow. Overall, 16% of all Intake patients completed care in the CPCs. Separately, the proportion of patients who did not meet Supertrack criteria and required transfer to a main ED treatment room remained stable at 14% during both study periods.

For the primary outcome, the roomed-to-disposition time for all Intake patients decreased from 88 minutes in the comparison period to 75 minutes during the intervention period, meeting the project aim. Intake Supertrack patients, who preferentially completed care in CPCs, experienced a more substantial reduction, with times decreasing from 152 to 77 minutes. Both measures demonstrated statistically significant centerline shifts on control charts and exceeded the target goal (Figs. 3A, B). A total of 729 Supertrack patients were evaluated in Intake during this period, resulting in a cumulative time savings of 911 patient-care hours.

**Fig. 3.**
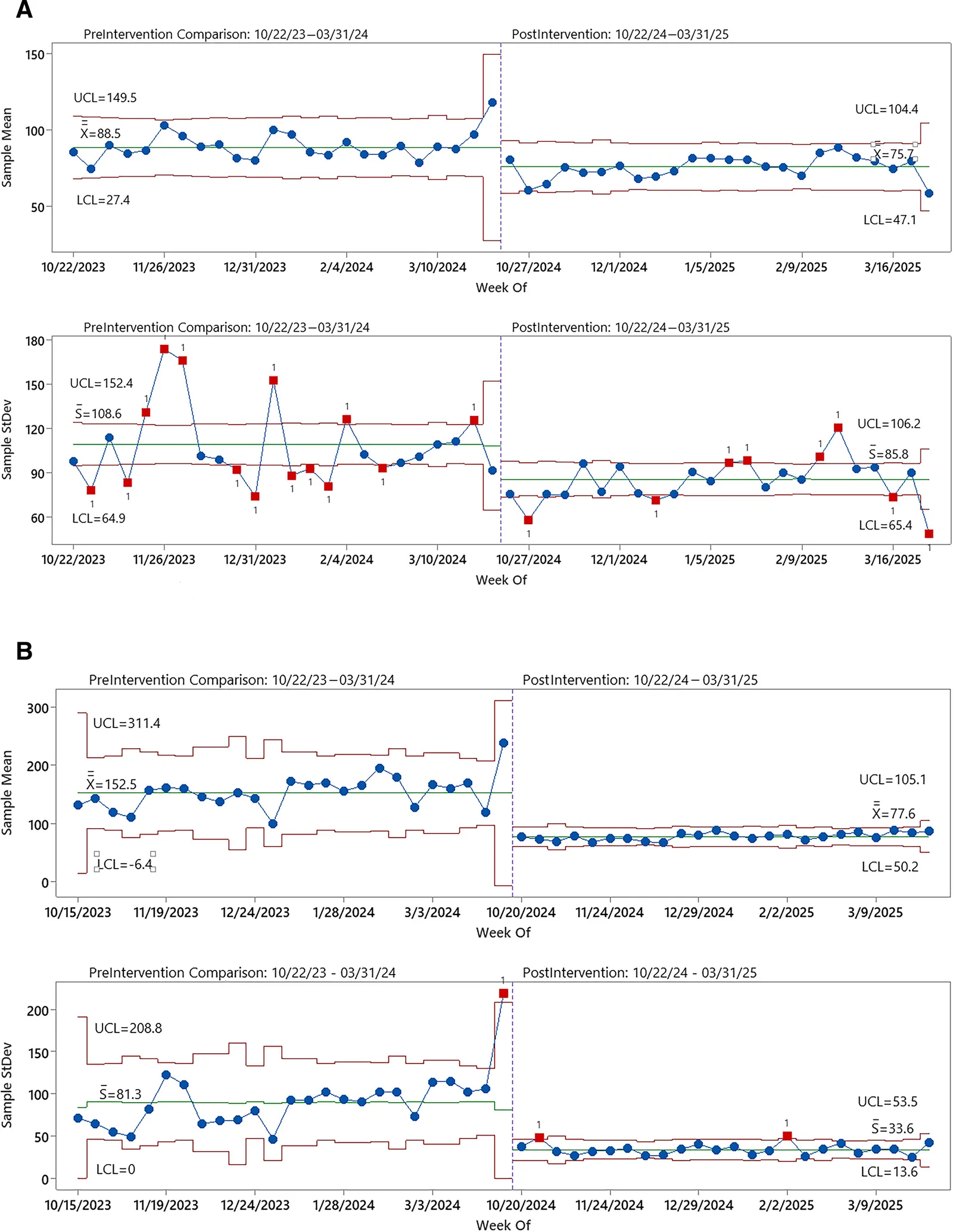
Average roomed-to-disposition time. A, Average roomed-to-disposition time for all Intake patients. B, Roomed-to-disposition time for Intake Supertrack patients.

The primary process measure, average time in the first room, decreased from 61 to 47 minutes for all Intake patients (Fig. 4) and from 64 to 48 minutes for Supertrack patients. The balancing measure demonstrated no increase in 72-hour return ED visits, which decreased from 5% preintervention to 3% postintervention.

**Fig. 4.**
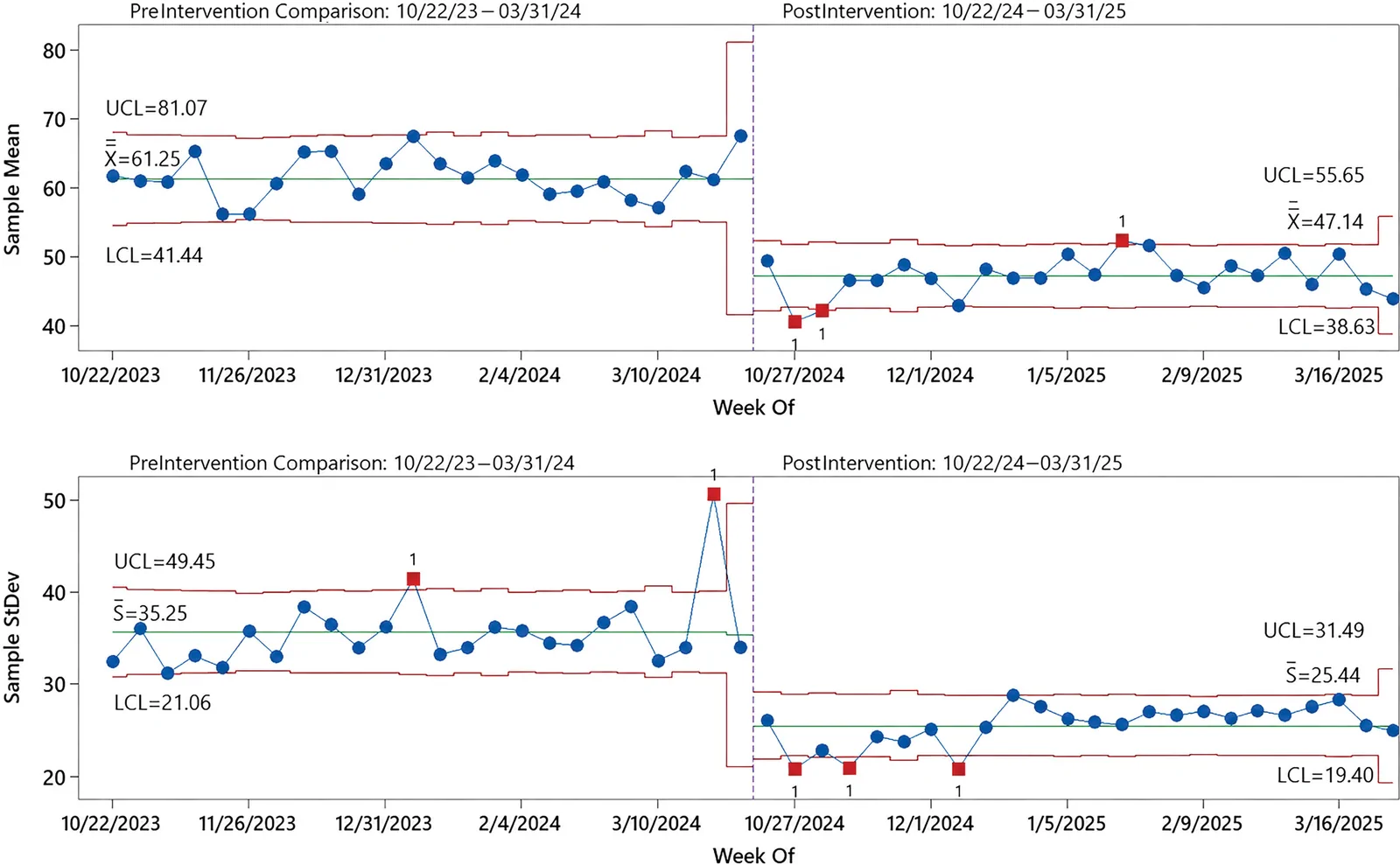
Average time in the first room for all Intake patients.

Secondary measures also improved following the implementation of the CPCs and associated Intake workflow changes. Average LOS for all Intake patients decreased from 213 minutes in the comparison period to 150 minutes in the intervention period, and from 286 to 148 minutes for Supertrack patients. LOS for all ED patients also decreased, from 285 to 227 minutes. Average arrival-to-provider time for Intake patients decreased from 105 to 66 minutes. The percent of LWBS for all ED patients declined from 6% to 3%.

## DISCUSSION

To improve patient flow through our Intake front-end process, we established CPCs as an alternative care space at the ED front end, streamlined workflows, and redefined team roles and responsibilities. These changes enabled faster disposition of appropriate patients directly at the front end, improving our roomed-to-disposition time for all Intake patients from 88 minutes in the previous respiratory season to 75 minutes during our intervention period. Intake Supertrack patients experienced a more substantial reduction, with times decreasing from 152 to 77 minutes, a savings of 75 minutes. Additionally, 16% of Intake patients completed care in CPCs, many of whom would have been transferred to a main ED treatment room under the previous Intake workflow.

Our quality improvement initiative demonstrated that thoughtful reorganization of existing ED Intake processes, without additional staffing, physical construction, or expansion, can meaningfully improve patient throughput, provider efficiency, and overall ED performance.

Our intervention centered on introducing CPCs to enable front-end completion of care, supported by repurposing Intake rooms for defined evaluation and intervention functions, and standardizing the responsibilities of physicians, RNs, and EMTs. These initiatives led to measurable improvements in throughput. We observed a 22% reduction in time spent in the first room, suggesting adherence to the new workflow and more efficient care.

Our primary outcome was the roomed-to-disposition time for patients seen in Intake, as this provides the most accurate reflection of throughput within the front-end process. We observed the most significant improvement among patients in the Supertrack pathway, with a 52% reduction in roomed-to-disposition time. Figure 3A represents all Intake patients, a heterogeneous patient population, and captures the operational variability inherent in a busy ED. As expected, some residual process variation remained following implementation. Nevertheless, overall variation decreased, as demonstrated by lower and narrower control limits and a statistically significant improvement in process capability. The more narrowly defined Supertrack cohort demonstrated substantially tighter process control following implementation (Fig. 3B), consistent with the primary intervention.

We also noted an increase in the number of patients designated as Supertrack. Before this intervention, physicians were often reluctant to assign patients to Supertrack, as it typically required a handoff to a provider in the main ED, which frequently had limited capacity. As a result, patients were often held in the Intake area to avoid a handoff, tying up rooms and delaying the evaluation of new patients. With the implementation of the new process and the ability to complete care within the front end, providers now feel more comfortable designating patients as Supertrack, with care progressing in the designated CPC spaces. This allows for improved flow and more efficient use of space throughout the ED.

This improvement also correlated with decreased LOS and LWBS among both Intake and nonintake patients, supporting a broad, department-wide effect of the intervention. Although other studies have demonstrated reductions in LWBS, we believe our interventions not only achieved similar results but also ensured that, beyond an initial medical screening examination, all patients seen received a comprehensive evaluation and completed care during their visit, enhancing both safety and quality of care. Although there were early apprehensions that an emphasis on visit efficiency and reduction in visit time would adversely affect patient care, 72-hour return rates remained stable throughout the intervention. These findings suggest that the intervention had no observed negative impact on short-term outcomes compared with preintervention processes.

There were initial concerns that the increased workload for RNs, EMTs, and physicians in the Intake area would affect efficiency and sustainability. The addition of the CPCs increased the number of patients assigned to Intake physicians and staff. It also increased the responsibility to alert physicians when results were available or observation periods were completed. Specifically, EMTs expressed hesitation due to the added responsibilities and uncertainty about the benefits. To address this, we provided regular data reviews with staff, which demonstrated sustained improvements in roomed-to-disposition times, LOS, and LWBS. Over time, this transparency and demonstrated success led to growing staff buy-in and engagement. EMTs specifically reported that the new process allowed them to function more consistently at the top of their scope.

Physicians working in Intake expressed strong support for the updated process. They value the ability to advance patient care without requiring a traditional room and appreciate being able to complete care at the front end without transferring patients to another provider in the main ED. Despite increased demands on providers and staff, we maintained stable patient volumes at the front end, underscoring the process’s efficiency and sustainability. There was initial concern that transitioning patients to CPCs could negatively impact patient and family satisfaction. However, no patient complaints related to the new process were received during the study period. General feedback from families reflected appreciation for the overall shorter visit duration.

Importantly, we achieved these improvements without any construction, large physical space changes, or an increase in staffing. This underscores the strength of process redesign and operational efficiency in addressing ED capacity challenges. Capacity and staffing constraints are common challenges across pediatric EDs, and, given their low resource requirements, these changes may be feasible for adoption by other institutions facing similar challenges.

There are limitations to this project. Our findings are specific to systems with an existing front-end Intake process and may be challenging to generalize to all ED settings. Additionally, in lower volume settings or EDs without such capacity constraints, there may be no need to implement alternative care spaces. Finally, during periods of reduced patient census, the urgency to use alternative care spaces may decline, potentially affecting sustainability. Continued evaluation and adjustment will be necessary to maintain process fidelity and staff engagement during such periods.

## CONCLUSIONS

As EDs continue to face rising volumes and space constraints, our experience suggests that operational innovations, rather than costly structural changes, can have a substantial impact. Future efforts should focus on sustaining gains during periods of lower volume and exploring how similar approaches can be adapted to different ED settings.

## ACKNOWLEDGMENTS

The authors thank the University of Colorado Institute for Healthcare Quality, Safety and Efficiency (IHQSE) Quality Improvement Writing Group for assisting with article preparation. The authors used ChatGPT (OpenAI, San Francisco, CA) solely for grammatical editing and proofreading assistance. The authors were responsible for all content and interpretations, and approved the final article.
